# Temperature Control of Nonlinear Continuous Stirred Tank Reactors Using an Enhanced Nature-Inspired Optimizer and Fractional-Order Controller

**DOI:** 10.3390/biomimetics11020153

**Published:** 2026-02-19

**Authors:** Serdar Ekinci, Davut Izci, Aysha Almeree, Vedat Tümen, Veysel Gider, Ivaylo Stoyanov, Mostafa Jabari

**Affiliations:** 1 Department of Computer Engineering, Bitlis Eren University, 13100 Bitlis, Turkey; sekinci@beu.edu.tr (S.E.); vtumen@beu.edu.tr (V.T.); 2 Department of Electrical and Electronics Engineering, Bursa Uludag University, 16059 Bursa, Turkey; davutizci@uludag.edu.tr; 3 Faculty of Engineering, İstanbul Aydın University, 34295 Istanbul, Turkey; ayshaalmeree@stu.aydin.edu.tr; 4 Distance Education Application and Research Center, Batman University, 72100 Batman, Turkey; veysel.gider@batman.edu.tr; 5 Department of Electrical Power Engineering, University of Ruse, 7017 Ruse, Bulgaria; 6 Faculty of Electrical Engineering, Sahand University of Technology, Tabriz 51335, Iran; m_jabari97@sut.ac.ir

**Keywords:** artificial lemming algorithm, joint opposite selection, temperature management, continuous stirred tank reactor, fractional-order controller

## Abstract

The temperature regulation of nonlinear continuous stirred tank reactor (CSTR) processes remains a challenging control problem due to strong nonlinearities, time-delay effects, and sensitivity to disturbances and parameter variations. Conventional proportional–integral–derivative (PID)-based control strategies often fail to provide the robustness and precision required under such conditions, motivating the use of more flexible controller structures and advanced optimization techniques. In this study, an enhanced joint-opposition artificial lemming algorithm (JOS-ALA) is proposed for the optimal tuning of a fractional-order PID (FOPID) controller applied to CSTR temperature control. The proposed JOS-ALA incorporates a joint opposite selection mechanism into the original ALA to improve population diversity, convergence stability, and resistance to local optima stagnation. A nonlinear CSTR model is linearized around a stable operating point, and the resulting model is employed for controller design and optimization. The FOPID controller parameters are tuned by minimizing a composite cost function that simultaneously accounts for tracking accuracy, overshoot suppression, and instantaneous error behavior. The effectiveness of the proposed approach is assessed through extensive simulation studies and benchmarked against state-of-the-art and high-performance metaheuristic optimizers, including ALA, electric eel foraging optimization (EEFO), linear population size reduction success-history based adaptive differential evolution (L-SHADE), and the improved artificial electric field algorithm (iAEFA). The benchmarking set is further extended with the success rate-based adaptive differential evolution variant (L-SRTDE) to broaden the comparative evaluation. Simulation results demonstrate that the JOS-ALA-based FOPID controller consistently achieves superior performance across multiple criteria. Specifically, it attains the lowest mean cost function value of 0.1959, eliminates overshoot, and yields a normalized steady-state error of 4.7290 × 10^−4^. In addition, faster transient response and improved robustness under external disturbances and measurement noise are observed when compared with competing methods. Statistical reliability of the observed performance differences is additionally examined using a Wilcoxon signed-rank test conducted over 25 independent runs. The resulting *p*-values confirm that the improvements achieved by the proposed approach are statistically significant at the 5% level across all pairwise algorithm comparisons. These findings indicate that the proposed JOS-ALA provides an effective and reliable optimization framework for high-precision temperature control in nonlinear CSTR systems and offers strong potential for broader application in complex process control problems.

## 1. Introduction

The regulation of nonlinear continuous stirred tank reactor (CSTR) processes represents a long-standing challenge in process control engineering due to their complex nonlinear dynamics, inherent time delays, strong parameter coupling, and high sensitivity to external disturbances. These characteristics frequently impair temperature regulation accuracy, reduce process efficiency, and may even compromise operational safety under varying operating conditions [1,2,3]. Consequently, the development of robust and high-precision control strategies for CSTR temperature management continues to be an active and important research area.

Traditionally, CSTR temperature control has been addressed using classical proportional–integral–derivative (PID) controllers, owing to their simple structure, ease of implementation, and widespread industrial acceptance [4,5]. Despite these advantages, classical PID controllers often struggle to achieve satisfactory performance in nonlinear and time-delay-dominated processes such as CSTRs. In practice, this limitation manifests in the form of excessive overshoot, prolonged settling times, and degraded robustness when system parameters or operating conditions deviate from their nominal values [6,7]. These shortcomings have motivated extensive research into more flexible controller structures capable of coping with the inherent complexity of CSTR dynamics.

Among the proposed alternatives, fractional-order PID (FOPID) controllers have attracted considerable attention as a powerful generalization of conventional PID control. By introducing fractional orders of integration and differentiation, FOPID controllers provide additional tuning degrees of freedom, enabling finer adjustment of system dynamics and improved robustness against nonlinearities and uncertainties [8,9]. These properties make FOPID controllers particularly suitable for complex thermal processes such as CSTR systems. However, the increased dimensionality of the FOPID parameter space significantly complicates the tuning process, rendering classical analytical or heuristic tuning approaches insufficient for achieving optimal performance.

To overcome this challenge, metaheuristic optimization algorithms have been increasingly adopted for tuning FOPID controllers. Such algorithms are well suited for navigating high-dimensional, nonlinear, and multimodal search spaces. A variety of metaheuristic techniques have been reported in the literature, including the artificial lemming algorithm (ALA) [10], electric eel foraging optimization (EEFO) [11], linear population size reduction success-history based adaptive differential evolution (L-SHADE) [12], and the improved artificial electric field algorithm (iAEFA) [13]. These methods have demonstrated strong global search capabilities and promising performance in controller tuning problems. Nevertheless, many existing metaheuristics still suffer from limitations such as premature convergence, insufficient population diversity, and stagnation in local optima, particularly when applied to complex control-oriented optimization landscapes.

Recent studies have shown that intelligent optimization-based control strategies can significantly enhance performance in nonlinear thermal and industrial systems [14,15]. Applications have been reported for electric furnaces, continuous stirred tank heater (CSTH) systems, and switched reluctance motors, where metaheuristic-tuned controllers achieved notable improvements in transient response, robustness, and disturbance rejection [16,17]. In this context, Alzakari et al. [18] proposed an enhanced cooperation search algorithm for tuning a FOPID controller applied to a nonlinear steam condenser system, demonstrating improved convergence speed and reduced overshoot. More generally, numerous optimization-based control frameworks have been developed for different nonlinear systems [19,20], many of which exhibit dynamic characteristics closely related to those encountered in CSTR temperature control.

Parallel to application-oriented studies, significant research efforts have focused on improving the core mechanisms of metaheuristic algorithms themselves. Jabari and Rad [21] employed the dung beetle optimizer (DBO) and ant lion optimizer (ALO) to tune PID and FOPID controllers for switched reluctance motor applications, achieving improved time-domain performance and reduced ripple effects. Zhao et al. [11] introduced the EEFO algorithm, inspired by the social foraging behavior of electric eels, and demonstrated its strong convergence performance on benchmark functions and engineering optimization problems. Of particular relevance to the present work, Arini et al. [22] proposed the joint opposite selection (JOS) mechanism, which combines selective leading opposition and dynamic opposition strategies to enhance the balance between exploration and exploitation in metaheuristic algorithms. When embedded into the Harris hawks optimization framework, JOS was shown to significantly improve convergence stability and solution quality.

Several studies have specifically investigated advanced control strategies for CSTR systems. Ibrahim [1] analyzed PID, two-degree-of-freedom PID, and model predictive control schemes for nonlinear CSTR processes, reporting improved performance for advanced controllers while relying on classical tuning techniques. Izci et al. [4] proposed a two-degree-of-freedom PID acceleration controller optimized using the starfish optimization algorithm (SFOA) for CSTH temperature regulation, achieving enhanced tracking accuracy and robustness at the expense of increased computational complexity. Xiao et al. [10] introduced the ALA and demonstrated its strong convergence and robustness through extensive benchmarking on CEC2017 and CEC2022 test functions; however, its applicability to practical nonlinear process control systems such as CSTRs was not explored. Other intelligent control approaches, including fuzzy-based wavelet neural network controllers [23] and coot bird optimization algorithm (CBOA)-based PID and FOPID tuning for CSTR temperature control [24], further confirm the effectiveness of optimization-driven strategies in managing complex thermal processes.

Although a wide range of nature-inspired optimizers has been reported in the literature, many of these methods exhibit recurring limitations when applied to control-oriented optimization problems, such as premature convergence, loss of population diversity, and stagnation in local optima. These issues become particularly pronounced in high-dimensional and strongly nonlinear search spaces, such as those arising from FOPID tuning for CSTR systems. The ALA already offers a balanced exploration–exploitation structure through its energy-guided behavioral transitions; however, its performance may still degrade in highly multimodal landscapes. The recently introduced JOS mechanism provides a principled means of addressing these shortcomings by combining selective leading opposition, which intensifies exploitation around promising regions, with dynamic opposite learning, which expands the effective search domain. Embedding JOS into ALA therefore yields an optimizer that preserves the adaptive dynamics of the original algorithm while systematically enhancing diversity and escape capability. This hybridization is particularly well suited to the present problem, where both global exploration and stable convergence are essential for reliable FOPID tuning in nonlinear, delay-dominated CSTR processes.

Motivated by the above observations, this study proposes an enhanced joint-opposition artificial lemming algorithm (JOS-ALA), in which the joint opposite selection mechanism is integrated into the original ALA framework to improve population diversity, convergence speed, and robustness against local optima stagnation. The proposed JOS-ALA is employed to optimally tune a FOPID controller for the temperature control of a linearized CSTR model. The effectiveness of the proposed approach is evaluated through comprehensive simulation studies and benchmarked against both state-of-the-art algorithms (ALA and EEFO) and high-performance optimizers (L-SHADE and iAEFA). In addition, a recent success rate-based adaptive differential evolution variant (L-SRTDE) is also included in the comparative analysis to further extend the benchmarking scope and provide a broader performance reference across modern adaptive differential evolution frameworks. The results demonstrate that the JOS-ALA-based FOPID controller achieves superior performance, including a mean cost function value of 0.1959, zero overshoot, and a normalized steady-state error of 4.7290 × 10^−4^, highlighting its potential as a robust and precise optimization framework for nonlinear process control applications.

The main contributions of this study can be summarized as follows:

- An enhanced variant of the artificial lemming algorithm, termed JOS-ALA, is proposed by embedding a joint opposite selection mechanism that integrates selective leading opposition and dynamic opposite strategies, thereby improving population diversity, convergence stability, and resistance to local optima stagnation.
- A FOPID controller is employed for CSTR temperature regulation, providing additional degrees of freedom that enable finer shaping of transient and steady-state dynamics in nonlinear and delay-dominated processes.
- A unified JOS-ALA-based tuning framework is developed for optimizing the five FOPID parameters under a composite performance index that simultaneously accounts for tracking accuracy, overshoot suppression, and instantaneous error behavior.
- A comprehensive comparative evaluation is conducted against both state-of-the-art and high-performance metaheuristics (ALA, EEFO, L-SHADE, and iAEFA), demonstrating the statistical and dynamic superiority of the proposed method.
- The comparative framework is further strengthened by including the L-SRTDE optimizer and by performing a Wilcoxon signed-rank statistical significance test over 25 independent runs to validate the reliability of pairwise performance differences.
- Extensive simulations under ideal and non-ideal conditions (including external disturbances and measurement noise) are performed, confirming the robustness, fast convergence, zero overshoot, and high-precision tracking capability of the proposed control strategy.

The remainder of this paper is organized as follows. Section 2 reviews the ALA, including its biological inspiration and optimization principles. Section 3 introduces the proposed JOS-ALA and describes its enhancements over the original algorithm. Section 4 presents the nonlinear CSTR process and its linearized modeling. Section 5 details the proposed control methodology, including the FOPID controller structure, cost function formulation, and JOS-ALA-based tuning framework. Section 6 provides comprehensive simulation results, extended comparative analyses including L-SRTDE benchmarking, and nonparametric statistical validation using the Wilcoxon signed-rank test under both ideal and non-ideal operating conditions. Finally, Section 7 concludes the paper and outlines potential directions for future research.

## 2. Artificial Lemming Algorithm

This section presents the mathematical foundation and operational workflow of the artificial lemming algorithm (ALA) [10]. The method is inspired by the adaptive survival strategies of lemmings in nature, which exhibit four dominant behaviors: long-distance migration, digging burrows, foraging for food, and evading natural predators. These behaviors are abstracted into complementary exploration and exploitation mechanisms that enable ALA to navigate complex search spaces efficiently. The algorithm integrates stochastic motion, Lévy flight, and an energy-regulated transition mechanism to dynamically balance global exploration and local refinement.

### 2.1. Initialization

ALA is a population-based optimizer. Let N denote the population size and Dim the problem dimension. The positions of all search agents are arranged in a matrix:

(1) $$Z=\left[\begin{array}{cccc} z_{1,1} & z_{1,2} & \cdots & z_{1,Dim}\\ z_{2,1} & z_{2,2} & \cdots & z_{2,Dim}\\ \vdots & \vdots & \ddots & \vdots \\ z_{N,1} & z_{N,2} & \cdots & z_{N,Dim} \end{array}\right]$$

where $z_{i,j}$ represents the j-th decision variable of the i-th lemming. Each element is initialized uniformly within the prescribed bounds.

(2) $$z_{i,j}=LB_{j}+rand\times (UB_{j}-LB_{j}),i=1,2,\ldots ,N,\;j=1,2,\ldots ,Dim$$

Here, $LB_{j}$ and $UB_{j}$ denote the lower and upper limits of the j-th dimension, and rand∈(0,1) is a uniformly distributed random number. At each iteration, the best-performing individual is identified and denoted by $Z_{best}$.

### 2.2. Long-Distance Migration (Exploration)

When resources are scarce, lemmings migrate over long distances to locate more favorable habitats. This behavior is modeled as a global exploration operator that combines Brownian motion with interactions among individuals.

(3) $$Z_{i}(t+1)=Z_{best}(t)+F\times BM\times [R\times (Z_{best}(t)-Z_{i}(t))+(1-R)\times (Z_{i}(t)-Z_{a}(t))]$$

In this expression, BM is a vector sampled from a standard normal distribution, modeling Brownian motion; $R\in [-1,1{]}^{1\times Dim}$ is generated as:

(4) R=2×rand(1,Dim)−1

and $Z_{a}$ is a randomly selected individual from the population. The scalar F∈{−1,+1} is defined by:

(5) $$F=\left\{\begin{array}{cc} +1, & \lfloor 2\times rand+1\rfloor =1\\ -1, & \lfloor 2\times rand+1\rfloor =2 \end{array}\right.$$

which randomly reverses the movement direction to enhance diversity. This operator enables agents to traverse distant regions while maintaining a loose attraction toward the current best solution.

### 2.3. Digging Holes (Exploration)

Lemmings excavate burrows to form safe shelters and access new resources. This localized exploration is modeled as:

(6) $$Z_{i}(t+1)=Z_{i}(t)+F\times L\times (Z_{best}(t)-Z_{b}(t))$$

where $Z_{b}$ is a randomly chosen individual and L∈(0,1) is an iteration-dependent coefficient.

(7) $$L=rand\times \left(1+sin\left(\frac{t}{2}\right)\right)$$

This mechanism perturbs the current position along a direction defined by the relative location of the best and a random peer, enabling the algorithm to probe nearby regions while retaining stochastic variability.

### 2.4. Foraging for Food (Exploitation)

During foraging, lemmings intensively search within their immediate habitat. A spiral movement is adopted to simulate this behavior:

(8) $$Z_{i}(t+1)=Z_{best}(t)+F\times spiral\times rand\times Z_{i}(t)$$

with

(9) spiral=radius×(sin(2π×rand)+cos(2π×rand))

(10) $$radius=\sqrt{\sum_{j=1}^{Dim}(z_{best,j}(t)-z_{i,j}(t){)}^{2}}$$

The radius corresponds to the Euclidean distance between the agent and the current best solution, defining the scope of local search. This operator intensifies exploitation around promising regions and improves convergence precision.

### 2.5. Evading Natural Predators (Exploitation)

When threatened, lemmings perform abrupt and deceptive movements. This behavior is modeled using Lévy flight:

(11) $$Z_{i}(t+1)=Z_{best}(t)+F\times G\times Levy(Dim)\times (Z_{best}(t)-Z_{i}(t))$$

where the escape coefficient G decreases over time.

(12) $$G=2\times \left(1-\frac{t}{T_{max}}\right)$$

The Lévy flight step is computed as:

(13) $$Levy(x)=0.01\times \frac{u\times \sigma }{|v{|}^{1/\beta }}$$

(14) $$\sigma ={\left(\frac{\Gamma (1+\beta )sin(\pi \beta /2)}{\Gamma ((1+\beta )/2)\beta 2^{(\beta -1)/2}}\right)}^{1/\beta }$$

with u,v∈(0,1) and β=1.5. Lévy motion introduces occasional long jumps, allowing the algorithm to escape local optima even during late iterations.

### 2.6. Energy-Guided Transition from Exploration to Exploitation

To regulate the dynamic balance between global exploration and local exploitation, ALA introduces an energy factor that emulates the gradual depletion of lemmings’ activity over time. This factor governs the behavioral switch among the four movement strategies. The energy function is defined as:

(15) $$E(t)=4\times arctan\left(1-\frac{t}{T_{max}}\right)\times ln\left(\frac{1}{rand}\right)$$

where t denotes the current iteration, $T_{max}$ is the maximum number of iterations, and rand∈(0,1) is a uniformly distributed random variable. For analytical convenience, an auxiliary variable is introduced:

(16) $$\delta =1-\frac{t}{T_{max}}$$

which allows the energy expression to be rewritten as follows.

(17) $$E(t)=4\times \arctan\;(\delta )\times ln\left(\frac{1}{rand}\right)$$

The stochastic nature of E(t) ensures that its value fluctuates while decreasing in expectation as the iterations proceed. The probability that the energy exceeds unity is evaluated as:

(18) $$P(E(t)>1)=\int_{0}^{1}\int_{0}^{e^{-\frac{1}{4arctan(\delta )}}}\;dr\;d\delta \approx 0.506$$

indicating that the likelihood of activating exploration and exploitation remains nearly equal around the threshold E(t)=1. This probabilistic symmetry yields a smooth and non-abrupt transition between phases.

## 3. Proposed Joint Opposite Selection-Based ALA

Although the original ALA already integrates multiple biologically inspired behaviors, its performance can still be hindered by premature convergence in highly multimodal landscapes or by insufficient diversification during later iterations. To overcome these limitations, a joint opposite selection (JOS) mechanism [22] is embedded into ALA, yielding an enhanced variant termed JOS-ALA.

**Figure 1:**
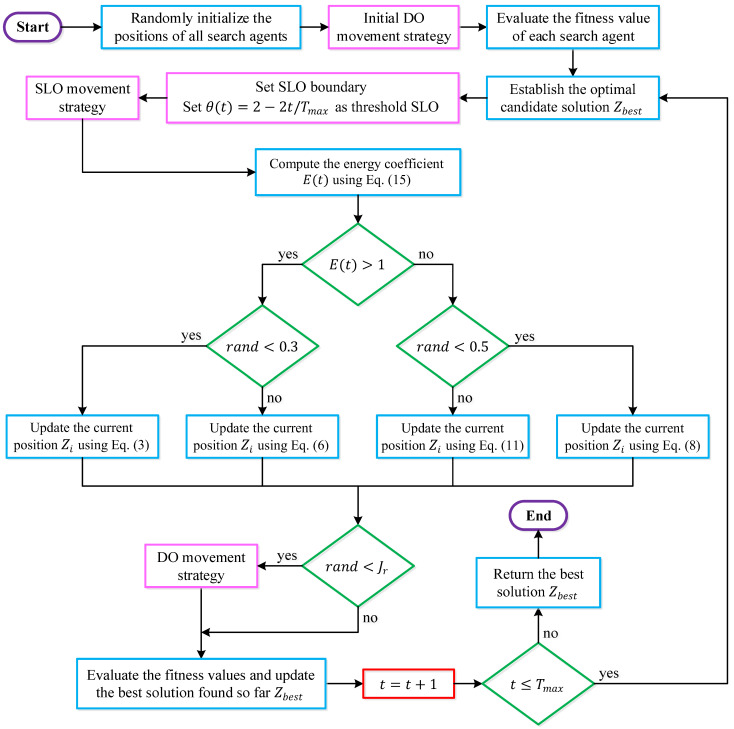
Detailed flowchart of proposed JOS-ALA.

The JOS strategy is derived from the joint application of two complementary opposition-based learning operators: selective leading opposition (SLO) and dynamic opposite (DO). These operators are philosophically and operationally designed to reinforce exploitation and exploration, respectively. The detailed flowchart of the proposed JOS-ALA is provided in Figure 1.

Selective leading opposition modifies only the eligible close dimensions of a search agent relative to the current best solution. Let $Z_{best}$ be the global best position and $Z_{i}$ the position of the i-th agent. For each dimension j, $dd_{j}=\left|z_{best,j}-z_{i,j}\right|$. A linearly decreasing threshold is defined as $\theta (t)=2-\left(2t/T_{max}\right)$. Dimensions satisfying $dd_{j}\leq \theta (t)$ are classified as close; otherwise, they are treated as far. The associativity between $Z_{i}$ and $Z_{best}$ is then evaluated using Spearman’s rank correlation coefficient.

(19) $$src=1-\frac{6\sum_{j=1}^{Dim}dd_{j}^{2}}{Dim(Dim^{2}-1)}$$

If src≤0 and the number of close dimensions exceeds the number of far dimensions, opposition is applied only on the close dimensions:

(20) $$z_{i,j}^{new}=LB_{j}+UB_{j}-z_{i,j},j\in D_{c}$$

where $D_{c}$ denotes the set of close dimensions. This mechanism selectively perturbs promising components of a solution, thereby intensifying exploitation without disrupting the global structure of the candidate. In JOS-ALA, SLO is executed after each main position update when E(t)≤1, i.e., during the exploitation-dominated phase. The threshold θ(t) naturally aligns with the energy decay of ALA, enabling synchronized intensification as convergence approaches.

Dynamic opposite aims to expand the reachable search region by generating asymmetric opposition moves. For a given position Z, the opposite point is first computed as $Z^{op}=LB+UB-Z$. A random reflection point is then generated as $Z^{r}=rand\times Z^{op}$ and the dynamic opposite position is obtained by $Z^{do}=Z+rand\times (Z^{r}-Z)$. This operator is activated probabilistically under a jumping rate $J_{r}$ (empirically set to $J_{r}=0.25$ in the literature. In JOS-ALA, DO is invoked:

- Once at initialization to diversify the starting population;
- During iterations when $rand<J_{r}$, particularly under E(t)>1, to reinforce exploration.

## 4. Mechanism of Continuous Stirred Tank Reactor

**Figure 2:**
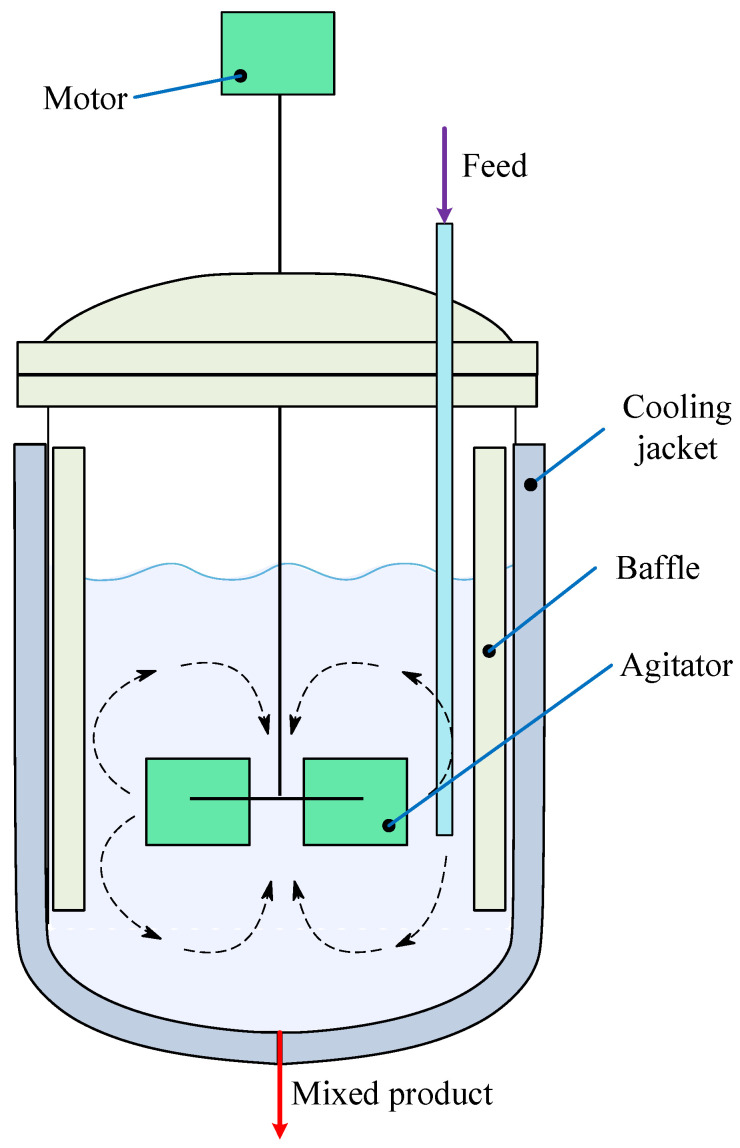
Cross-sectional diagram of a CSTR.

A continuous stirred tank reactor (CSTR) is widely used in the chemical process industry for conducting liquid-phase reactions under continuous flow conditions. The reactor ensures uniform mixing and temperature distribution through continuous agitation and heat exchange, typically facilitated by an external jacket that allows thermal regulation using a heating or cooling fluid. Figure 2 illustrates a cross-sectional view of the CSTR, showing key components such as the inflow and outflow streams, mixing impeller, and the surrounding thermal jacket [25].

The dynamic behavior of the reactor system is governed by a set of nonlinear differential equations derived from fundamental mass and energy balances. These equations describe the evolution of reactant concentration and reactor temperature over time, considering the thermal interaction with the jacket fluid. To facilitate controller design, the nonlinear model is linearized around a stable operating point defined by a reactant concentration of CA=0.98molm3, reactor temperature T=304.2 K, and jacket temperature $T_{j}=280\;K.$

To enable a simplified yet accurate representation of the system dynamics for control purposes, the process is approximated using a stable first-order plus time delay (SFOPTD) model. This linear model is obtained via the Sundaresan and Krishnaswamy method [26], which identifies the best-fit first-order plus dead-time approximation from the step response data. Specifically, a 10 K step input is applied to the jacket temperature, and the resulting temperature change in the reactor is analyzed. The identified transfer function relating the jacket temperature input $T_{j}$, to the reactor temperature output T, is given as follows [24]:

(21) $$G_{cstr}\left(s\right)=\frac{0.85}{0.4355s+1}e^{-0.0135s}$$

This transfer function captures the essential dynamic characteristics of the reactor system, including the process gain, time constant, and inherent delay. It serves as the foundation for subsequent control strategy development and optimization.

## 5. Proposed Method

This section describes the architecture of the proposed control strategy, which integrates a fractional-order PID (FOPID) controller with the hybrid JOS-ALA optimization algorithm. The goal is to ensure high-performance control of the nonlinear CSTR process under a variety of operating conditions.

### 5.1. Controller

**Figure 3:**
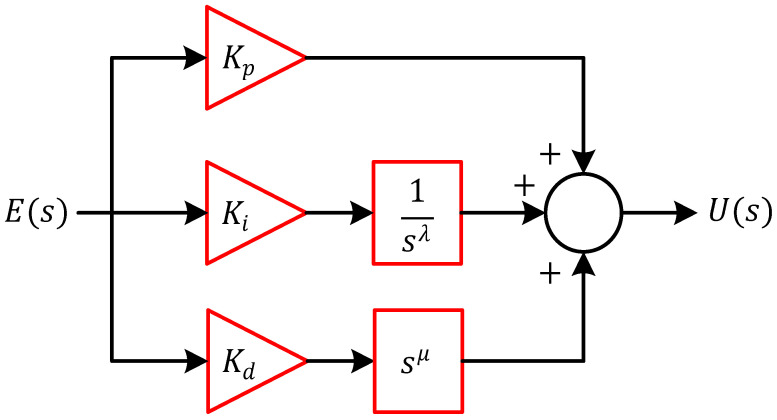
Block diagram of FOPID controller.

At the core of the proposed method is the FOPID controller, a generalization of the classical PID that offers additional flexibility by introducing two fractional orders of integration and differentiation. Its transfer function is defined as:

(22) $$FOPID\left(s\right)=K_{p}+\frac{K_{i}}{s^{\lambda }}+K_{d}s^{\mu }$$

where $K_{p}$, $K_{i}$, $K_{d}$, λ, μ and s are, the proportional gain, the integral gain, the derivative gain, the fractional order of integration, the fractional order of differentiation and the Laplace variable, respectively. Compared to conventional PID controllers, the FOPID structure provides two extra degrees of freedom λ and μ enabling more precise shaping of the controller’s frequency and time-domain response. This additional flexibility is especially beneficial for controlling complex, nonlinear, and time-varying systems like CSTR. The block diagram representation of the FOPID controller is shown in Figure 3, where the fractional operators are typically realized using approximation methods such as Oustaloup’s recursive filters.

### 5.2. Cost Function and Parameter Limits

To evaluate the performance of each candidate FOPID parameter set, a scalar cost function is defined as follows:

(23) $$IAE=\int_{0}^{t_{f}}|e\left(t\right)|dt$$

(24) $$CF=\sigma \times IAE+\left(1-\sigma \right)\times n_{os}$$

where CF is the cost function, IAE is the integral of absolute error performance metric, $n_{os}$ is the normalized overshoot and σ=0.85 is the weighting factor. Here, r(t) denotes the reference temperature signal (setpoint) applied to the CSTR, while y(t) represents the measured reactor temperature at the system output. The tracking error is defined as e(t)=r(t)−y(t) over a time horizon $t_{f}=2$ s. This cost function enables simultaneous optimization of both transient and steady-state performance. Adjusting σ, allows for flexible prioritization: for instance, a high σ, emphasizes tracking precision, while a low σ, focuses on minimizing overshoot, which is especially important in safety-critical or sensitive process applications like chemical reactors. The adopted cost function is deliberately formulated to capture the multi-faceted nature of temperature control in CSTR systems. The integral of absolute error (IAE) term penalizes the cumulative tracking deviation over the entire simulation horizon, thereby promoting fast convergence and minimal steady-state error. This term ensures that long-lasting residual errors are strongly discouraged. The normalized overshoot component explicitly constrains excessive transient excursions, which are particularly undesirable in chemical reactors due to safety and product quality considerations. By incorporating overshoot directly into the objective, aggressive control actions that may yield fast responses at the expense of process stability are systematically penalized. The instantaneous error term introduces sensitivity to abrupt deviations, enabling the optimizer to suppress sharp transient spikes that may arise from disturbances or model uncertainties. Together, these components provide a balanced representation of both transient and steady-state behavior. The weighting factor σ governs the relative emphasis between tracking accuracy and overshoot suppression. A larger α favors rapid and precise tracking, whereas a smaller value prioritizes smooth and conservative responses. This formulation allows the tuning process to be tailored to the operational requirements of the CSTR, where both precision and safety are critical.

To ensure realistic and stable control behavior, the FOPID parameters are constrained within the following practical bounds: $1\leq K_{p},K_{i}\leq 100$, $0.001\leq K_{d}\leq 2$, 0.5≤λ,μ≤1.5. These limits are selected based on domain expertise and prior knowledge of the system dynamics. They are designed to prevent actuator saturation, instability, or overly aggressive control actions. Constraining the parameter search space also improves the efficiency of the optimization algorithm by reducing computational effort and avoiding infeasible or suboptimal regions.

### 5.3. Implementation

**Figure 4:**
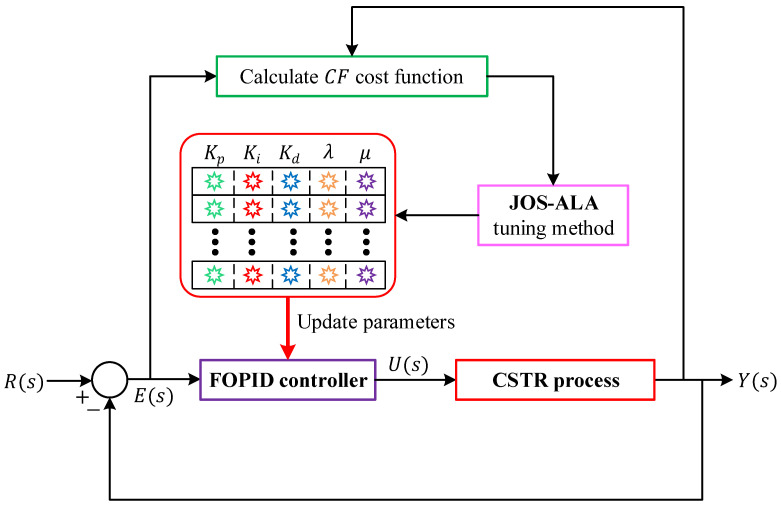
Implementation of the proposed JOS-ALA to tune FOPID controller for CSTR process.

The implementation of the proposed framework follows the closed-loop architecture illustrated in Figure 4, in which the optimization and control tasks are tightly integrated through a dual-loop structure. The inner loop performs real-time temperature regulation of the CSTR, whereas the outer loop is responsible for the adaptive tuning of the FOPID controller parameters by the JOS-ALA. As shown in the figure, the reference signal R(s) is continuously compared with the measured reactor temperature Y(s) to generate the tracking error E(s). This error is processed by the FOPID controller, yielding the control signal U(s), which drives the CSTR process. The resulting output is fed back to the summing junction, thereby forming a conventional closed-loop control system. At this level, the controller operates with a fixed set of parameters.

Surrounding this control loop, an outer optimization loop is established by the JOS-ALA tuning module. For each candidate parameter set generated by the optimizer, the closed-loop response of the CSTR is simulated. The resulting output trajectory Y(s) is used to compute the composite cost function, which encapsulates tracking accuracy, overshoot behavior, and instantaneous error characteristics. This evaluation step is represented in Figure 4 by the “Calculate CF cost function” block. Based on the computed cost value, the JOS-ALA updates the population of candidate solutions and generates a refined set of controller parameters. These updated parameters are then injected into the FOPID controller through the “Update parameters” path, replacing the previous gains and fractional orders. The closed-loop system is subsequently re-evaluated using the new parameter set. This iterative interaction continues until the termination criterion of the optimizer is satisfied. Through this structure, the controller tuning process is performed directly within the closed-loop environment of the CSTR. Each candidate solution is assessed under identical dynamic conditions, ensuring that the optimization is guided by the true control performance rather than by surrogate or decoupled metrics. The integration of JOS-ALA as an outer loop allows the FOPID parameters to be progressively refined while preserving the stability and physical meaning of the control loop. Consequently, the final parameter set corresponds to a controller that is optimally adapted to the dynamic behavior of the reactor and robust against the nonlinear and delay-dominated characteristics of the CSTR process.

## 6. Comparative Simulation Results

### 6.1. Adopted Algorithms for Comparisons

To rigorously assess the effectiveness of the proposed JOS-ALA-based FOPID tuning framework, a comprehensive comparative study was carried out against a set of well-established and high-performance metaheuristic optimization algorithms. The comparison set was deliberately chosen to include both contemporary nature-inspired methods and algorithms that are widely recognized for their strong global optimization capability.

The first group consists of state-of-the-art bio-inspired optimizers. The ALA [27] represents the baseline method from which the proposed approach is derived and is known for its adaptive behavior in dynamic search environments. The EEFO algorithm [28], which integrates evolutionary principles with fuzzy mechanisms, is included due to its reported ability to enhance convergence speed while preserving solution diversity. The second group comprises high-performance optimizers that have demonstrated remarkable effectiveness across a wide range of benchmark and engineering problems. L-SHADE [12] is a refined variant of differential evolution that combines adaptive parameter control with a gradually shrinking population, yielding strong global search performance. The iAEFA [13] represents a recent enhancement of electric-field-based optimization, designed to maintain a careful balance between exploration and exploitation through adaptive elitism.

To ensure that the comparison is both fair and reproducible, all algorithms were evaluated under identical experimental conditions. Each optimizer employed a population of 30 individuals and was executed for a maximum of 100 iterations. Furthermore, every method was independently run 25 times to account for the stochastic nature of metaheuristic search and to enable statistically meaningful performance assessment. These settings were selected based on methodological considerations, existing literature, and preliminary sensitivity analyses. A population size of 30 and an iteration limit of 100 are commonly adopted in metaheuristic-based controller tuning studies and provide an effective compromise between exploration capability and computational cost for the five-dimensional FOPID search space. Smaller populations were found to limit diversity and increase the likelihood of premature convergence, whereas substantially larger populations offered only marginal performance improvements while incurring a considerable increase in runtime. Conducting 25 independent runs ensures reliable estimation of both central tendency and dispersion, thereby allowing robust statistical comparisons among the competing methods. In addition, the bounds imposed on the FOPID parameters were determined in accordance with domain expertise and ranges reported in related CSTR and thermal-process control studies. This guarantees physical realizability, numerical stability, and avoidance of excessively aggressive control actions. Collectively, these design choices establish a fair, consistent, and computationally tractable benchmarking environment for evaluating the proposed approach.

### 6.2. Statistical Analysis

**Figure 5:**
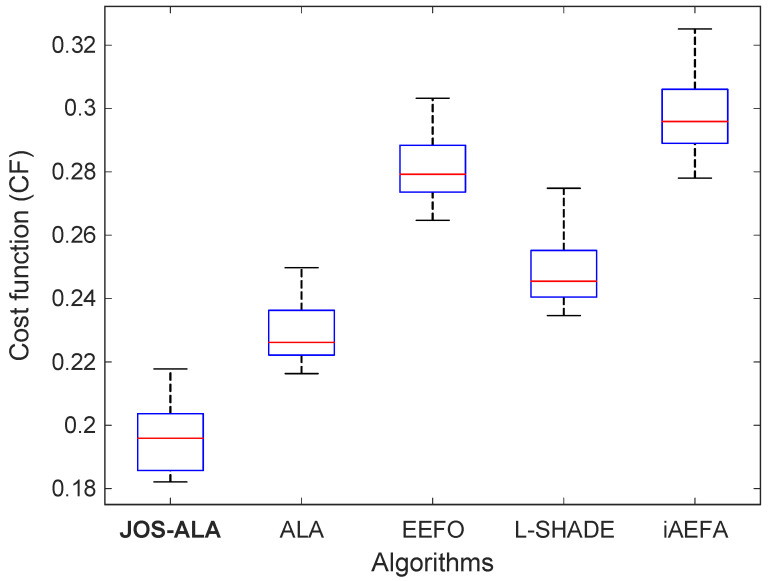
Comparative boxplot analyses for JOS-ALA, ALA, EEFO, L-SHADE and iAEFA.

To provide a more robust evaluation of optimization consistency and reliability, a statistical performance analysis was conducted over multiple independent runs. Figure 5 presents the boxplot comparison for the five-optimization algorithms: JOS-ALA, ALA, EEFO, L-SHADE, and iAEFA. Complementary to this, Table 1 summarizes their key statistical metrics, including mean, standard deviation (SD), best and worst performance, and overall rank.

As shown in the boxplots, the JOS-ALA achieves the most compact distribution of results, reflecting its strong repeatability and reduced sensitivity to random initialization. It consistently yields lower objective function values with minimal variation across runs. This is confirmed numerically in Table 1, where JOS-ALA achieves the lowest mean value (0.1959), the best individual performance (0.1821), and the top ranking overall. Its standard deviation (0.0102) is also low, indicating stable behavior. In contrast, algorithms such as iAEFA and EEFO show both higher mean values (0.2980 and 0.2810, respectively) and wider performance ranges. These larger spreads in their boxplots suggest greater variability and less predictable convergence behavior. Although ALA and L-SHADE perform better than EEFO and iAEFA, they still fall short of the consistency and optimality achieved by JOS-ALA. Overall, the results clearly highlight the statistical superiority of JOS-ALA, validating it as not only an effective but also a reliable optimization framework. Its ability to consistently produce high-quality solutions across multiple trials makes it a promising choice for real-world controller tuning applications, where both performance and robustness are critical.

### 6.3. Cost Function Minimization

**Figure 6:**
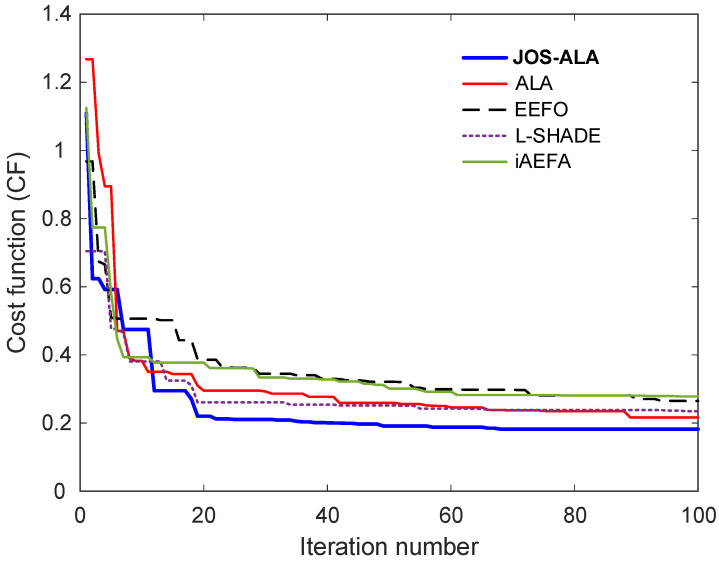
Comparative convergence curves for JOS-ALA, ALA, EEFO, L-SHADE and iAEFA.

To assess the optimization performance of the proposed method, Figure 6 presents the comparative convergence curves for JOS-ALA, ALA, EEFO, L-SHADE, and iAEFA during the cost function minimization process. This evaluation focuses on how effectively each algorithm reduces the objective function over successive iterations, which is critical for identifying high-quality controller parameters. As shown in the figure, the proposed JOS-ALA exhibits the most rapid and stable convergence behavior among all compared methods. It reaches a lower cost value within fewer iterations, indicating its superior efficiency in navigating the search space. The convergence curve of JOS-ALA not only descends more steeply in the early iterations but also demonstrates minimal fluctuations, suggesting a balanced exploration–exploitation trade-off.

In contrast, other methods such as EEFO and iAEFA show slower convergence and settle at higher final cost values, reflecting less effective optimization performance. The ALA and L-SHADE algorithms perform moderately well but still fall short of the precision and speed achieved by JOS-ALA. These findings underscore the effectiveness of the JOS-ALA hybrid optimization strategy in minimizing the cost function more reliably and efficiently than the other state-of-the-art techniques. The ability to consistently achieve lower cost values with faster convergence is a key advantage in practical controller design tasks where computational time and solution quality are critical.

### 6.4. Obtained Controller Parameters and Time Domain Performance Analyses

The controller parameters obtained through different optimization algorithms JOS-ALA, ALA, EEFO, L-SHADE, and iAEFA are summarized in Table 2. These parameters were specifically tuned to optimize the closed-loop behavior of the CSTR process, and they form the foundation for a fair and systematic performance comparison.

**Figure 7:**
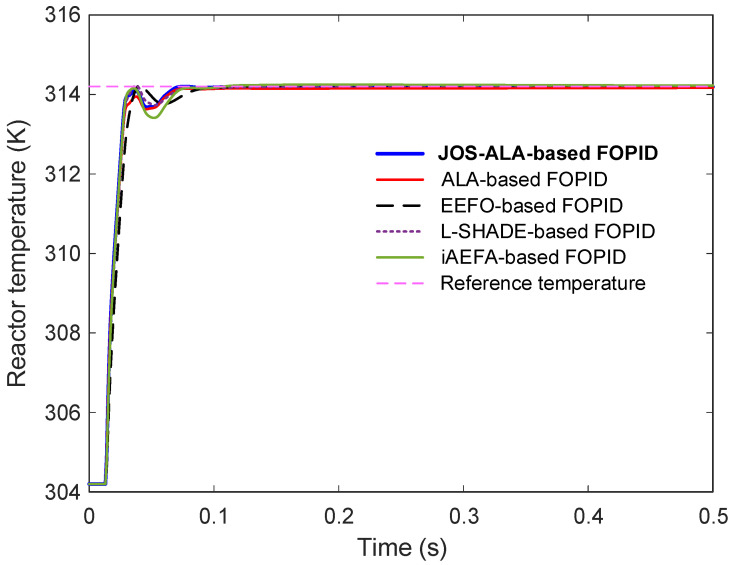
Comparative step response of JOS-ALA, ALA, EEFO, L-SHADE and iAEFA for reactor temperature.

**Figure 8:**
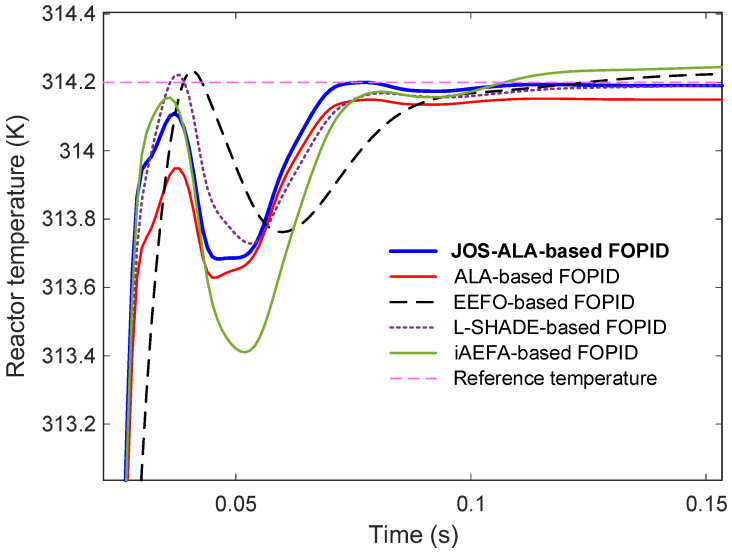
Enlarged view of Figure 7.

The comparative step responses for reactor temperature are illustrated in Figure 7, with an enlarged view provided in Figure 8 to highlight detailed behaviors around the transient period. From the results, it is clear that the JOS-ALA-based FOPID controller demonstrates superior performance compared to the other methods. Specifically, it achieves faster rise and settling times, minimal overshoot, and smoother convergence to the steady-state.

Quantitative performance metrics extracted from the time-domain responses are listed in Table 3. The proposed JOS-ALA-based FOPID achieves the best rise time (0.0126 s) and settling time (0.0619 s) among all tested approaches. Importantly, it maintains zero overshoot, ensuring system stability and preventing potential stress on the process components. Additionally, the JOS-ALA-based controller achieves the lowest steady-state error, as evidenced by a normalized value of $4.7290\times 10^{-4}$, highlighting its exceptional precision. In comparison, other methods such as ALA and iAEFA also show reasonable performance but exhibit higher steady-state errors or slight overshoots, which could degrade long-term system stability or efficiency in practical applications. Overall, these results confirm that the proposed JOS-ALA-based FOPID controller not only outperforms existing optimization-based approaches in key dynamic and steady-state metrics but also offers a robust and highly accurate solution for the temperature regulation of CSTR processes.

### 6.5. Computational Time Analysis

In addition to convergence behavior and control performance, the computational burden of each optimizer is an important practical consideration, particularly for controller tuning tasks that may be repeated or embedded in higher-level design workflows. All algorithms were executed under identical hardware and software conditions using MATLAB (2025b) on the same workstation. The average computational time per independent run was recorded over 25 trials. The mean execution times were measured as 68.6159 s for JOS-ALA, 59.6842 s for ALA, 65.4613 s for EEFO, 71.8960 s for L-SHADE, and 73.6615 s for iAEFA. Although the proposed JOS-ALA incurs a slightly higher computational cost than the original ALA, this increase is moderate and stems from the additional joint-opposition operations introduced to enhance population diversity and convergence stability. Notably, JOS-ALA remains computationally more efficient than L-SHADE and iAEFA, while delivering substantially superior solution quality and consistency. The observed trade-off between execution time and optimization accuracy is therefore favorable: the modest increase in runtime is compensated by significant gains in convergence reliability, reduced dispersion across runs, and improved closed-loop control performance. From a practical perspective, the tuning process is performed offline, and the resulting controller parameters are deployed in real time without further optimization overhead. Consequently, the reported computational cost does not hinder real-time operation and is well justified by the marked improvement in control precision and robustness achieved by the proposed method.

### 6.6. Performance Comparison L-SRTDE Algorithm

To further broaden the comparative evaluation, the proposed JOS-ALA-based FOPID tuning framework was also benchmarked against the success rate-based adaptive differential evolution (L-SRTDE) algorithm [14], a success-rate–driven adaptive differential evolution variant known for its strong global search capability and stable convergence characteristics. The L-SRTDE optimizer was applied to the same FOPID parameter tuning problem using identical controller bounds, cost function formulation, population size, iteration limit, and simulation environment. In this way, a consistent and fair performance comparison was ensured.

The statistical performance indicators obtained from multiple independent runs are summarized in Table 4. The reported metrics include mean objective value, standard deviation, best and worst values, and final ranking. It can be observed that both algorithms produce low-dispersion results, indicating reliable convergence behavior. However, the JOS-ALA method achieves a lower mean cost value (0.1959) than L-SRTDE (0.2020). A similar trend is observed for the best objective value, where JOS-ALA reaches 0.1821, while L-SRTDE attains 0.1901. Although the standard deviation of L-SRTDE is slightly smaller, the overall performance ranking favors JOS-ALA due to its superior mean and best-case results. Using the L-SRTDE algorithm, the optimized FOPID controller parameters were obtained as $K_{p}=24.3096$, $K_{i}=52.6269$, $K_{d}=0.1130$, λ=1.0053, and μ=1.0521. These parameters were then employed in the closed-loop CSTR temperature control simulation, and the resulting step response was compared with that of the JOS-ALA-based FOPID controller.

**Figure 9:**
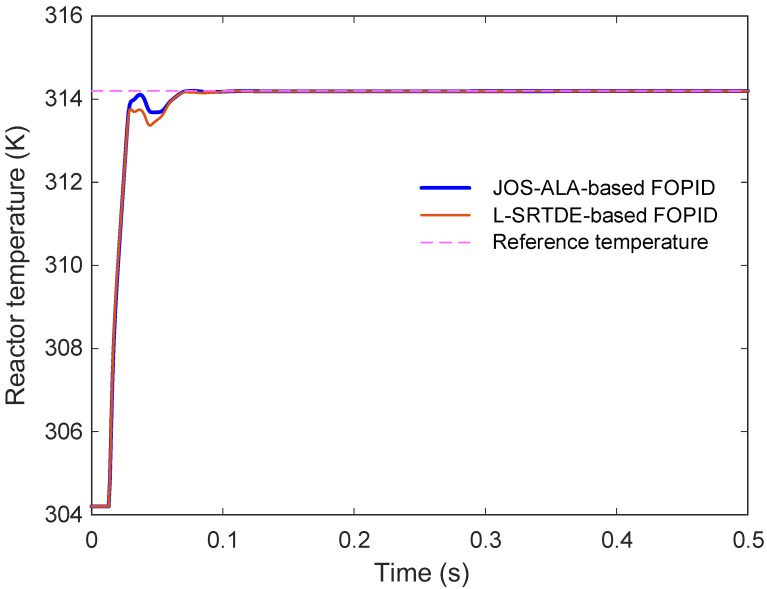
Comparative step response of JOS-ALA and L-SRTDE for reactor temperature.

The comparative reactor temperature responses are shown in Figure 9. It is observed that both controllers provide rapid reference tracking and stable convergence toward the desired operating temperature. The JOS-ALA-based FOPID controller reaches the reference with a slightly smoother transient profile and without observable overshoot. In contrast, the L-SRTDE-based design exhibits a small transient overshoot and a marginally more pronounced deviation during the early response phase. After the transient interval, both responses converge closely to the reference temperature, indicating satisfactory steady-state behavior in both cases.

A quantitative comparison of the time-domain performance metrics is provided in Table 5, including rise time, settling time, percentage overshoot, and normalized steady-state error. The rise times of the two controllers are nearly identical, measured as 0.0126 s for JOS-ALA and 0.0128 s for L-SRTDE. The settling times are likewise very close, with values of 0.0619 s and 0.0621 s, respectively. A clearer distinction appears in the overshoot and steady-state accuracy measures. The JOS-ALA-based controller maintains zero overshoot, whereas the L-SRTDE-based controller produces a small overshoot of 0.0052%. Moreover, the normalized steady-state error achieved by JOS-ALA remains significantly lower. These results indicate that, while L-SRTDE delivers competitive dynamic performance, the proposed JOS-ALA framework provides improved transient smoothness and higher steady-state precision under the same tuning objective. Overall, the extended comparison demonstrates that both optimizers are capable of producing high-quality FOPID parameter sets for the CSTR temperature control problem. Nevertheless, lower average cost, zero-overshoot response, and improved steady-state accuracy are consistently achieved with the JOS-ALA-based design.

### 6.7. Wilcoxon Signed-Rank Test

In addition to descriptive statistics and boxplot-based dispersion analysis, a nonparametric statistical significance study was conducted using the Wilcoxon signed-rank test [29]. This test is widely adopted for pairwise comparison of stochastic optimization algorithms when normal distribution of results cannot be guaranteed. Since each optimizer in this study was executed over 25 independent runs under identical conditions, the Wilcoxon signed-rank framework provides an appropriate basis for assessing whether the observed performance differences are statistically meaningful. The test was performed between the proposed JOS-ALA and each competing method, namely ALA, EEFO, L-SHADE, iAEFA, and L-SRTDE, using the per-run objective function values. The null hypothesis assumes that the median performance difference between two paired algorithms is zero. A significance level of 0.05 was adopted.

The Wilcoxon signed-rank test results are summarized in Table 6, where the *p*-values and comparison outcomes are reported. For all pairwise comparisons, the computed *p*-values are below the selected significance threshold. In particular, extremely small *p*-values (1.2290 × 10^−5^) are obtained for the comparisons against ALA, EEFO, L-SHADE, and iAEFA, indicating very strong statistical evidence in favor of performance differences. For the comparison between JOS-ALA and L-SRTDE, the *p*-value is 0.0370, which also remains below 0.05, confirming statistical significance. Across all tested pairs, the winner indicator favors JOS-ALA. This outcome demonstrates that the improvements achieved by the proposed optimizer are not only numerically superior in terms of mean objective value and response metrics, but also statistically significant with respect to run-to-run variability. The Wilcoxon analysis therefore supports the reliability and consistency of the proposed method and complements the earlier statistical indicators and graphical analyses. Here, the indicator h=1 denotes rejection of the null hypothesis at the 5% significance level.

### 6.8. Comparative Performance Analyses with Respect to Reported Approaches

**Figure 10:**
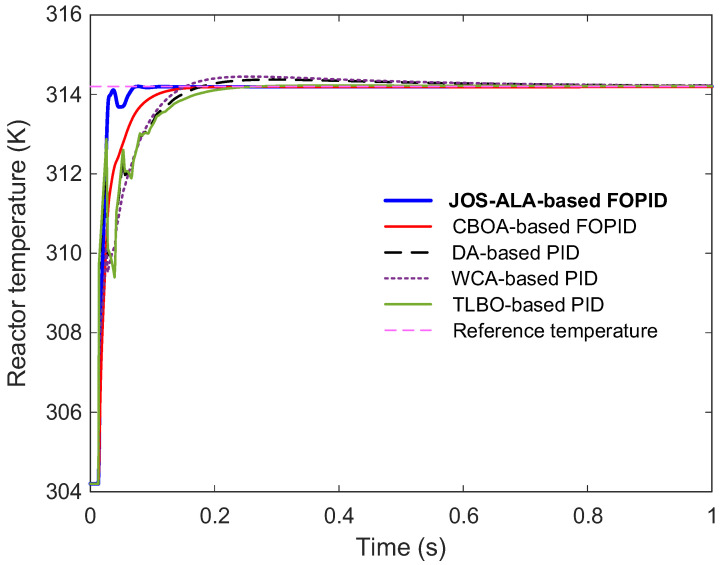
Comparative step response with respect to reported approaches.

To further highlight the effectiveness of the proposed control approach, a comparative performance analysis was conducted against several well-established methods from the literature, namely CBOA-based FOPID, DA-based PID, WCA-based PID, and TLBO-based PID controllers [24]. The controller parameters obtained for each method are listed in Table 7, providing a basis for fair comparison. The step response comparison is depicted in Figure 10. It is evident that the proposed JOS-ALA-based FOPID controller significantly outperforms the other methods in terms of transient behavior. The system under the proposed controller achieves a faster rise time and settles more quickly, while also exhibiting minimal overshoot and smooth convergence to the steady-state. These advantages are critical for enhancing the responsiveness and stability of the controlled process.

Table 8 further quantifies the comparative performance using standard time-domain metrics, including rise time, settling time, percentage overshoot, and steady-state error. The JOS-ALA-based FOPID controller achieves the best results across all metrics. Specifically, it demonstrates the lowest rise time (0.0126 s) and settling time (0.0619 s), indicating its superior dynamic response. Furthermore, it maintains zero percent overshoot and an exceptionally small steady-state error of $4.7290\;\times \;10^{-4}$, highlighting its high precision and stability. Compared to other reported methods, the proposed approach offers a substantial improvement in both dynamic and steady-state performance. These results affirm the proposed controller’s capability to deliver fast, stable, and accurate control, making it a highly promising solution for practical applications requiring high-performance operation.

### 6.9. Input Signal Tracking Performance

**Figure 11:**
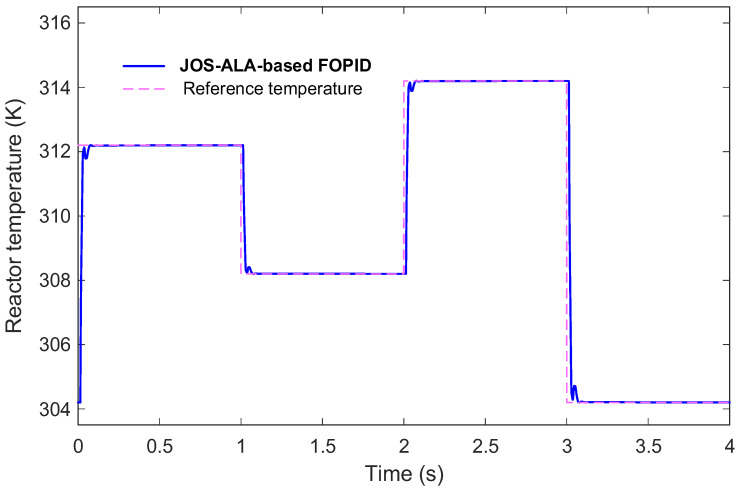
Input signal tracking performance of the proposed method.

The input signal tracking capability of the proposed control method was assessed and is presented in Figure 11. This figure illustrates the system’s response in tracking the reference input signals over time. As shown, the proposed method exhibits a high degree of accuracy in following the desired input trajectories. The control inputs smoothly and promptly adjust to changes in the reference signal without significant delay or oscillation. Importantly, the tracking errors remain minimal throughout the entire operation period, highlighting the effectiveness of the control design.

Moreover, the transitions between different reference levels are handled gracefully, with no evidence of abrupt or unstable behavior. This smooth and precise tracking performance underlines the method’s ability to achieve reliable control action, which is crucial for practical applications where accurate input following directly impacts overall system stability and efficiency. Overall, the results confirm that the proposed method ensures excellent input signal tracking, further validating its applicability for real-world control tasks where precision and robustness are essential.

### 6.10. Performance Evaluation for Non-Ideal Conditions

**Figure 12:**
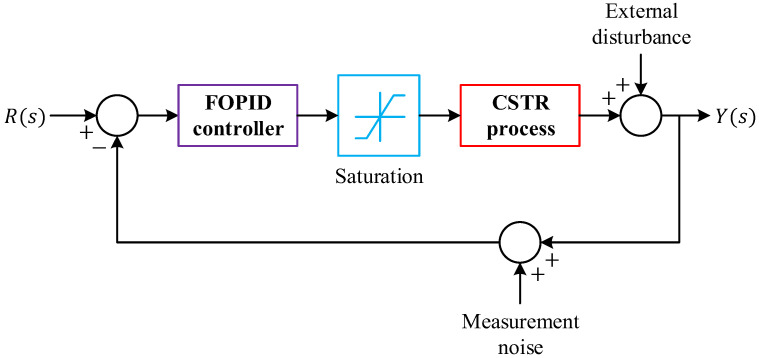
Non-ideal model of CSTR process.

In this study, measurement noise is not filtered or preprocessed prior to control action. Instead, it is directly injected into the feedback signal to emulate realistic sensor imperfections commonly encountered in industrial CSTR installations. This design choice allows the robustness of the proposed JOS-ALA–tuned FOPID controller to be evaluated under adverse and unmitigated conditions. By exposing the controller to noisy measurements during closed-loop operation, the tuning process implicitly accounts for noise-induced fluctuations through the composite cost function, particularly via the instantaneous error and IAE terms. As a result, the optimizer favors parameter sets that maintain stable and smooth responses despite corrupted feedback, rather than relying on external filtering mechanisms. This approach ensures that the reported robustness reflects the intrinsic resilience of the control strategy itself. To further validate the robustness of the proposed control approach, we evaluated its performance under non-ideal conditions, including external disturbances and measurement noise. Figure 12 illustrates the modified CSTR process model incorporating these non-ideal elements, thereby creating a more realistic testing environment for the controller.

**Figure 13:**
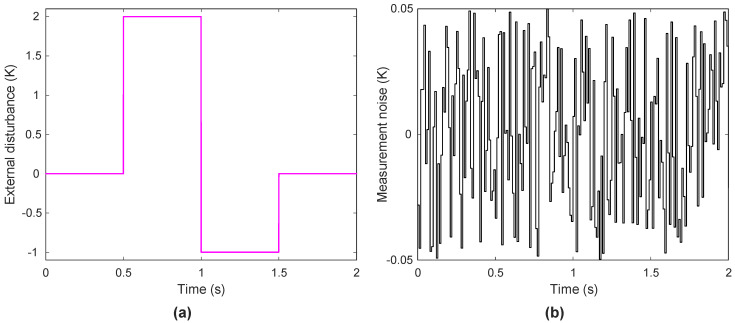
Adopted external disturbance (**a**) and measurement noise (**b**).

The adopted external disturbance and measurement noise profiles featured in Figure 13 demonstrate their introduction into the system. Operational fluctuations were emulated through the disturbances while measurement noise represented real sensor inaccuracies. These conditions serve as fundamental benchmarks to evaluate both the robustness and practical applicability of the control strategy.

**Figure 14:**
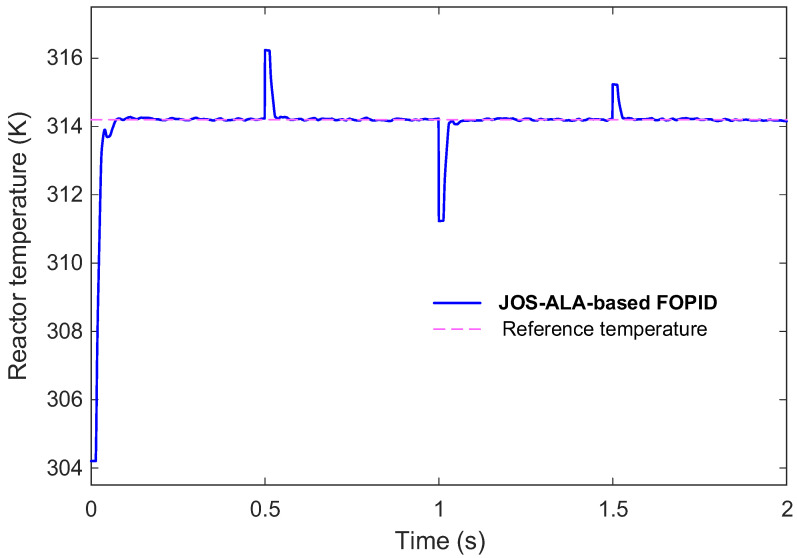
Performance of the proposed JOS-ALA based FOPID approach under non-ideal conditions.

The system’s response under these non-ideal conditions is depicted in Figure 14. As observed, despite the presence of significant external disturbances and measurement noise, the proposed approach maintained excellent performance. The process variables closely tracked their desired reference values, demonstrating strong disturbance rejection capabilities. Furthermore, the system exhibited fast convergence, minimal overshoot, and stable behavior throughout the simulation period. These results confirm that the proposed control method is not only effective under ideal scenarios but also robust against typical real-world uncertainties. Such characteristics make it highly suitable for practical deployment in industrial CSTR operations where disturbances and measurement noise are inevitable.

## 7. Conclusions

In this study, an enhanced ALA, JOS-ALA was proposed for the optimal tuning of a FOPID controller applied to the temperature regulation of nonlinear CSTR processes. The primary objective was to address the limitations of conventional PID-based control strategies and standard metaheuristic optimizers when confronted with the strong nonlinearities, time-delay characteristics, and disturbance sensitivity inherent in CSTR systems. The proposed JOS-ALA framework was developed by integrating a joint opposite selection mechanism into the original ALA, thereby improving population diversity, convergence stability, and resistance to local optima stagnation. The enhanced optimizer was employed to tune the five parameters of the FOPID controller for a linearized CSTR model obtained around a stable operating point. Comprehensive simulation studies were conducted to evaluate the effectiveness of the proposed approach and to ensure a fair comparison with both state-of-the-art metaheuristics (ALA and EEFO) and high-performance optimization algorithms (L-SHADE and iAEFA). The comparative study was further extended with the L-SRTDE algorithm to broaden the adaptive differential evolution benchmark reference. The comparative results clearly demonstrated the superiority of the JOS-ALA-based FOPID controller across multiple performance criteria. Statistically, the proposed method achieved the lowest mean cost function value of 0.1959 with minimal variance, indicating strong consistency and robustness across independent runs. Additional nonparametric statistical analysis based on the Wilcoxon signed-rank test confirmed that the observed performance improvements over all compared optimizers, including L-SRTDE, are statistically significant at the 5% significance level. From a control performance perspective, it delivered faster transient responses, zero overshoot, and an exceptionally small normalized steady-state error of 4.7290 × 10^−4^. Furthermore, the controller maintained reliable tracking accuracy and stable behavior under external disturbances and measurement noise, confirming its robustness in non-ideal operating conditions. When compared with previously reported optimization-based control approaches in the literature, the proposed method exhibited substantial improvements in both dynamic and steady-state performance metrics. Despite these promising results, certain limitations of the present study should be acknowledged. The controller design and optimization were based on a linearized representation of the nonlinear CSTR process, which may not fully capture the system dynamics under large operating variations. In addition, the computational complexity associated with the JOS-ALA may pose challenges for real-time or resource-constrained implementations. These aspects warrant further investigation to enhance the practical applicability of the proposed approach.

Future research may focus on extending the JOS-ALA framework to fully nonlinear CSTR models and experimental validation in real industrial environments. Further efforts may also be directed toward reducing computational overhead, exploring hybrid metaheuristic structures, and adapting the proposed optimizer to other complex nonlinear process control problems. Overall, the results presented in this work indicate that JOS-ALA constitutes a robust and effective optimization framework for high-precision temperature control and offers strong potential for broader adoption in intelligent process control applications aligned with modern Industry 4.0 requirements. The extended benchmarking and statistical significance analyses further reinforce the reliability and generalizability of the reported performance gains.

## Figures and Tables

**Table 1:** Statistical performance of JOS-ALA, ALA, EEFO, L-SHADE and iAEFA.

Algorithm	Mean	SD	Best	Worst	Rank
JOS-ALA	0.1959	0.0102	0.1821	0.2178	1
ALA	0.2300	0.0102	0.2163	0.2497	2
EEFO	0.2810	0.0102	0.2647	0.3032	4
L-SHADE	0.2492	0.0115	0.2346	0.2748	3
iAEFA	0.2980	0.0131	0.2780	0.3251	5

**Table 2:** Obtained controller parameters via JOS-ALA, ALA, EEFO, L-SHADE and iAEFA.

Control Method	$K_{p}$	$K_{i}$	$K_{d}$	λ	μ
JOS-ALA-based FOPID	25.0272	54.8040	0.1144	1.0014	1.0339
ALA-based FOPID	24.3960	46.6840	0.1104	0.9849	1.0359
EEFO-based FOPID	21.2946	55.0543	0.2783	0.9857	0.7792
L-SHADE-based FOPID	24.7851	58.9936	0.1237	1.0504	0.9996
iAEFA-based FOPID	23.0777	60.7810	0.2152	0.9990	0.9108

**Table 3:** Time domain response metrics achieved via JOS-ALA, ALA, EEFO, L-SHADE and iAEFA.

Control Method	$n_{rt}$ (s)	$n_{st}$ (s)	$n_{os}$ (%)	$n_{sse}$ (%)
JOS-ALA-based FOPID	0.0126	0.0619	0	4.7290 × 10^−4^
ALA-based FOPID	0.0132	0.0636	0	0.4778
EEFO-based FOPID	0.0159	0.0765	0.3587	0.1620
L-SHADE-based FOPID	0.0130	0.0653	0.2176	0.6971
iAEFA-based FOPID	0.0127	0.0680	0.4849	0.0159

**Table 4:** Statistical performance comparison of JOS-ALA and L-SRTDE optimizers for FOPID tuning.

Algorithm	Mean	SD	Best	Worst	Rank
JOS-ALA	0.1959	0.0102	0.1821	0.2178	1
L-SRTDE	0.2020	0.0070	0.1901	0.2148	2

**Table 5:** Time-domain performance metrics of JOS-ALA-based and L-SRTDE-based FOPID controllers.

Control Method	$n_{rt}$ (s)	$n_{st}$ (s)	$n_{os}$ (%)	$n_{sse}$ (%)
JOS-ALA-based FOPID	**0.0126**	**0.0619**	**0**	**4.7290 × 10^−4^**
L-SRTDE-based FOPID	0.0128	0.0621	0.0052	0.0525

**Table 6:** Wilcoxon signed-rank test results for pairwise comparison with JOS-ALA.

Algorithm	*p*-Value	h	Winner
JOS-ALA versus ALA	1.2290 × 10^−5^	1	JOS-ALA
JOS-ALA versus EEFO	1.2290 × 10^−5^	1	JOS-ALA
JOS-ALA versus L-SHADE	1.2290 × 10^−5^	1	JOS-ALA
JOS-ALA versus iAEFA	1.2290 × 10^−5^	1	JOS-ALA
JOS-ALA versus L-SRTDE	0.0370	1	JOS-ALA

**Table 7:** Controller parameters obtained via reported approaches in the literature.

Control Method	$K_{p}$	$K_{i}$	$K_{d}$	λ	μ
CBOA-based FOPID	16.334	36.206	0.168	0.964	0.898
DA-based PID	13.299	40.130	0.268	1	1
WCA-based PID	12.146	39.840	0.157	1	1
TLBO-based PID	13.936	32.297	0.278	1	1

**Table 8:** Comparative time domain metrics with respect to reported approaches.

Control Method	$n_{rt}$ (s)	$n_{st}$ (s)	$n_{os}$ (%)	$n_{sse}$ (%)
JOS-ALA-based FOPID	0.0126	0.0619	0	4.7290 × 10^−4^
CBOA-based FOPID	0.0476	0.1076	0	0.8627
DA-based PID	0.0833	0.1483	1.7114	0.0658
WCA-based PID	0.0775	0.3601	2.4750	0.0519
TLBO-based PID	0.0857	0.1704	0.2284	0.0505

## Data Availability

All produced data are available within the manuscript.
